# Real-world impact of transitioning from one lipoprotein(a) assay to another in a clinical setting

**DOI:** 10.1016/j.ajpc.2024.100726

**Published:** 2024-08-25

**Authors:** Janeni Jeevanathan, Sigrid M. Blom, Thomas Olsen, Kirsten B. Holven, Erik K. Arnesen, Torleif Trydal, Børge G. Nordestgaard, Michael Sovershaev, Ying Chen, Kjetil Retterstøl, Jacob J. Christensen

**Affiliations:** a Department of Nutrition, Institute of Basic Medical Sciences, University of Oslo, P.O. Box 1046 Blindern, 0317, Oslo, Norway; b Novartis Norway AS, Nydalen alle 37, 0484, Oslo, Norway; c Norwegian National Advisory Unit on Familial Hypercholesterolemia, Department of Endocrinology, Morbid Obesity and Preventive Medicine, Oslo University Hospital Aker, P.O. Box 4959 Nydalen, 0424, Oslo, Norway; d Department of Clinical Research, Sørlandet Hospital, SSHF, P.O. Box 416 Lundsiden, 4604, Kristiansand, Norway; e The Department of Clinical Biochemistry, Herlev and Gentofte Hospital, Copenhagen University Hospital, Borgmester Ib Juuls Vej 73, opgang 7, 2730, Herlev, Denmark; f Department of Clinical Medicine, Faculty of Health and Medical Sciences, University of Copenhagen, Blegdamsvej 3B, 2200 Copenhagen N, Denmark; g Fürst Medical Laboratory, P. O. Box 158 Alnabru, 0614, Oslo, Norway; h Oslo Metropolitan University, P. O. Box 4, Norway; i The Lipid Clinic, Department of Endocrinology, Morbid Obesity and Preventive Medicine, Oslo University Hospital Aker, P. O. Box 4959 Nydalen, 0424, Oslo, Norway

**Keywords:** Lipids, Lipoprotein(a), Lipoprotein(a) assay

## Abstract

**Background and aims:**

Different lipoprotein(a) [Lp(a)] assays may affect risk stratification of individuals and thus clinical decision-making. We aimed to investigate how transitioning between Lp(a) assays at a large central laboratory affected the proportion of individuals with Lp(a) result above clinical thresholds.

**Methods:**

We studied nationwide clinical laboratory data including 185,493 unique individuals (47.7 % women) aged 18-50 years with 272,463 Lp(a) measurements using Roche (2000-2009) and Siemens Lp(a) assay (2009-2019).

**Results:**

While the majority of individuals (66-75 %) had low levels of Lp(a) (<30 mg/dL) independent of the assay used, the Roche assay detected 20 % more individuals with Lp(a) >50 mg/dL, 40 % more individuals with Lp(a) >100 mg/dL and 80 % more individuals with Lp(a) > 180 mg/dL than the currently used Siemens assay, likely due to calibration differences.

**Conclusion:**

Transitioning from one Lp(a) immunoassay to another had significant impact on Lp(a) results, particularly in individuals approaching clinically relevant Lp(a) thresholds.

## Graphical abstract

**Figure:**
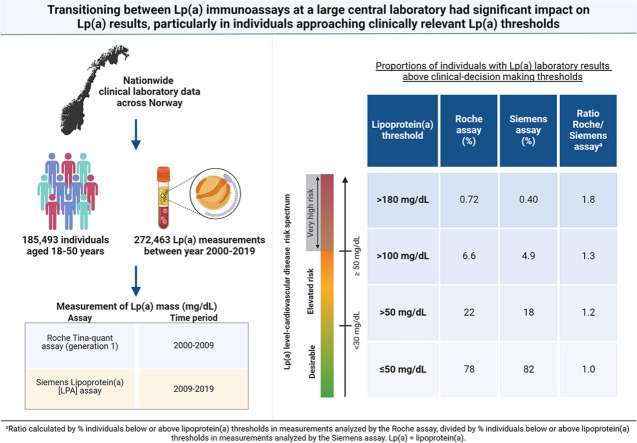

## 1 Introduction

Lipoprotein(a) [Lp(a)] is an apoB-containing low-density lipoprotein (LDL) particle with the glycoprotein apolipoprotein(a) [apo(a)] attached by a disulfide bond [1]. There is strong evidence for the role of high levels of Lp(a) as an independent and causal risk factor for atherosclerotic cardiovascular diseases (ASCVD) [2].

The genetic polymorphism which determines the composition of apo(a) makes it difficult to accurately measure Lp(a) levels [3]. Different assays have been used to analyze Lp(a) over time, and a secondary reference material was developed to assist in standardization of commercial Lp(a) assays, but this is unfortunately no longer available [3,4]. Despite these efforts, there is no gold standard immunoassay for measuring Lp(a) as of today. A few studies have evaluated the performance characteristics of Lp(a) assays and observed significant differences across assays [5,6]. Such findings might be due to the high degree of heterogeneity in size and composition of Lp(a), causing isoform-dependent bias and inaccurate calibrators [7].

Measuring Lp(a) is recommended by numerous clinical guidelines [8,9] and a consensus statement from the European Atherosclerosis Society states that “Lp(a) should be measured at least once in adults to identify those with high cardiovascular risk” [2]. However, there is a lack of studies on the clinical consequences of the use of different assays for measuring Lp(a).

Given that Lp(a) levels are determined mainly by genetics, it has been commonly believed that measuring Lp(a) once in a lifetime is sufficient; implicitly, even if conducted using a different assay many years ago. The initial measurement of an individual might have been conducted using an assay which is no longer commercially available. Nevertheless, it is likely that clinicians would consider historic Lp(a) measurements in risk prediction and therapeutic decision-making, and they are thus still clinically relevant.

Emerging new medications to lower Lp(a), along with the growing understanding of the risks associated with high Lp(a) levels, will likely lead to a substantial increase in Lp(a) measurements. Consequently, real-world data on the variability between major commercially used methods is important for evaluating whether an individual may benefit clinically from lowering Lp(a).

Using the coefficient of variation (CV) to express uncertainty poses significant challenges when measured values vary by more than 100-fold from the lowest to the highest. Additionally, a major proportion of the population (75 %) has low Lp(a) values [[10], [11], [12]] where even large measurement uncertainties have no clinical consequence. However, for individuals with high Lp(a) values, the opposite is true. The outdated perspective that Lp(a) values are simply low or high overlooks the crucial differences in risk associated with varying levels of elevated Lp(a).

In the high range of Lp(a) values, there is a significant disparity in risk between those with moderately elevated levels, which increase risk by 25 %, and those with significantly elevated levels, which can increase risk by two-fold or more [2]. In this high range, it is likely that specific cut-off values will be established based on cost-benefit analyses and the willingness to pay for new selective Lp(a)-reducing drugs. Hence, it is important to evaluate the variation both within new measurement methods and to explore the variation that occur when transitioning from an old method to a new one in a real-world situation.

Therefore, in the present study, using 272,463 Lp(a) measurements between 2000-2019 from the largest medical laboratory in Norway, we aimed to assess the real-world impact of transitioning between two different Lp(a) immunoassays. By focusing on the real-world impact, we sought to provide a nuanced understanding of how changes in assay methodology influence Lp(a) measurements and consequently patient management, notably in large cohorts in a clinical setting.

## 2 Materials and methods

### 2.1 Study population

The data was retrieved from the clinical laboratory database at Fürst Medical Laboratory (Fürst), the largest medical laboratory in Norway, covering most of the country with a preponderance of the south-eastern part. Fürst analyzes serum samples that are mainly ordered by general practitioners from the primary healthcare. The costs of Lp(a) measurements are covered by the health system financed by public service reimbursement, meaning that there is no financial bias regarding whether or not an individual receives Lp(a) measurement. For the present study, we included 185,493 unique individuals with 272,463 Lp(a) measurements between 2000 and 2019. Over 95 % of the serum Lp(a) measurements were analyzed within 24 h after sample collection, and the rest were analyzed within 3 days, aimed at keeping the specimens at 2-8 ^°^C. Because some lipid-lowering drugs can modestly alter plasma Lp(a) [13], the data material was restricted to individuals ≤50 years of age where fewer than 5 % use lipid-lowering drugs according to the Norwegian Prescription Database (Figure S1). Also, at this age cardiovascular disease and early death are unlikely to have prevented those with very high Lp(a) levels in getting a measurement.

The measurement is mainly carried out as part of a general health examination or on clinical indication. Therefore, it was important to assess whether this introduced significant bias in the clinical laboratory database at Fürst Medical Laboratory compared to the general population. There are no national data on the level of serum lipids in Norway; however, we compared the mean total cholesterol levels of the individuals within the study database with age and sex-adjusted participants from two large Norwegian population-based studies, the Trøndelag Health Study (HUNT) and Tromsø study, covering the same time period and geographical area as the Fürst database. Total cholesterol levels were slightly higher in the study database compared to HUNT, but were similar to the Tromsø study when considering the confidence intervals (5.0 mmol/L in the Tromsø study and 5.3 mmol/L (95 % confidence interval (CI): 4.8-5.8) in the study database in Northern Norway in women, and 5.4 and 5.8 mmol/L (95 % CI: 5.2-6.3) in men, respectively (Table S1). This implies that the study database is not much biased towards high cardiovascular risk individuals. With a distribution of Lp(a) resembling that in the Copenhagen General Population Study and the UK Biobank [12,14], our database seems to be representative for the general population with respect to Lp(a).

### 2.2 Measurement of Lp(a) and other biochemical variables

Lp(a) was analyzed by two differently calibrated assays throughout the period. From January 2000 to September 2009, the analyses were done by particle-enhanced immunoturbidimetric assay Lipoprotein(a) (Tina-quant) as provided by Roche Diagnostics (generation 1) (Mannheim, Germany) traceable to a highly purified Lp(a), the in-house master calibrator, on Modular P analyzer. From September 2009 to June 2018, the analyses were done by particle-enhanced immunoturbidimetric assay Lipoprotein(a) (LPA) as provided by Siemens Healthineers (Forchheim, Germany) traceable to an internal standard, on Advia 2400 Chemistry instrument. From June 2018 to December 2019 the Siemens assay were run on Advia Chemistry XPT instrument. The Siemens Lp(a) reagent was a Randox reagent traceable to the reference material WHO/IFCC SRM 2B. The method evaluation by the laboratory September 2009 found that the Siemens LPA assay had 50.6 % and 62.9 % lower values compared to the Roche assay at the levels of respectively 20 mg/dL and 80 mg/dL measured in 30 patient samples.

During the period, adjustments of the measurement intervals were performed. Measuring interval in 2000-2009 was 6.0-354 mg/dL. Measuring intervals in 2009-2016, 2016-2018 and 2018-2019 were 2.5-90 mg/dL, 10-85 mg/dL and 10-340 mg/dL, respectively. External quality assessment (EQA) from Labquality Helsinki, Finland, was performed throughout the period.

Other clinical chemistries included in Table 1 were measured simultaneously with the measurement of Lp(a) at Fürst Medical Laboratory in some of the individuals. The laboratory was accredited according to NS-EN ISO 15189 using published clinical chemistry methods. Some clinical chemistry methods were first introduced during the period of the study (2000-2019). Estimated Glomerular Filtration Rate (eGFR) was calculated from the 2009 CKD-EPI equation [15] using the isotope dilution mass spectrometry (IDMS)-traceable methods for the creatinine measurements, initially with the compensated Jaffe and later with an enzymatic assay.

**Table 1:** Patient characteristics of the study cohort based on biochemical variables measured in serum samples.

Lipoprotein(a) decile	N	Patient Characteristics			
		≤70th, N = 132,092	70th - 80th, N = 18,400	80th - 90th, N = 17,872	≥90th, N = 16,566
Lipoprotein(a) mg/dL	184,930	10 (6, 15)	39 (34, 44)	62 (56, 69)	90 (85, 112)
Sex	184,930				
Men		69,910 (53 %)	9,102 (49 %)	9,348 (52 %)	8,495 (51 %)
Women		62,182 (47 %)	9,298 (51 %)	8,524 (48 %)	8,071 (49 %)
Age, years	184,930	39 (31, 45)	39 (31, 45)	39 (31, 45)	40 (32, 45)
Triglycerides mmol/L	111,417	1.21 (0.84, 1.85)	1.17 (0.83, 1.75)	1.20 (0.84, 1.78)	1.23 (0.88, 1.82)
Cholesterol mmol/L	173,580	5.10 (4.40, 5.90)	5.20 (4.50, 6.00)	5.30 (4.60, 6.00)	5.60 (4.90, 6.40)
LDL-C mmol/L	163,275	3.18 (2.55, 3.90)	3.27 (2.64, 3.99)	3.36 (2.74, 4.05)	3.61 (2.96, 4.32)
LDL-C-corr mmol/L	163,275	3.09 (2.46, 3.81)	2.98 (2.36, 3.70)	2.89 (2.29, 3.60)	2.83 (2.19, 3.55)
HDL-C mmol/L	173,383	1.31 (1.09, 1.59)	1.31 (1.09, 1.60)	1.32 (1.10, 1.60)	1.34 (1.13, 1.61)
Apolipoprotein A1 g/L	33,759	1.50 (1.35, 1.67)	1.50 (1.35, 1.68)	1.50 (1.35, 1.69)	1.54 (1.39, 1.71)
Apolipoprotein B g/L	33,878	1.00 (0.83, 1.19)	1.03 (0.87, 1.23)	1.08 (0.91, 1.27)	1.18 (1.00, 1.37)
HbA1c %	52,899	5.30 (5.10, 5.60)	5.30 (5.10, 5.60)	5.30 (5.10, 5.60)	5.35 (5.10, 5.60)
Glucose mmol/L	54,475	5.00 (4.60, 5.40)	4.90 (4.60, 5.30)	4.90 (4.60, 5.30)	5.00 (4.60, 5.40)
Creatinine µmol/L	141,029	75 (65, 85)	75 (65, 86)	76 (66, 86)	76 (66, 87)
eGFR mL/min/1.73m^2^	141,029	78 (61, 93)	74 (60, 92)	78 (61, 93)	76 (61, 93)
TSH mU/L	136,206	1.47 (1.04, 2.04)	1.46 (1.03, 2.05)	1.48 (1.06, 2.04)	1.47 (1.06, 2.05)
Aspartate aminotransferase U/L	97,737	22 (18, 28)	22 (18, 28)	22 (18, 28)	23 (18, 28)
Alanine aminotransferase U/L	132,770	25 (18, 37)	24 (18, 36)	25 (18, 36)	25 (18, 36)
Gamma-glutamyl transferase U/L	95,701	21 (14, 35)	20 (14, 34)	21 (14, 34)	22 (15, 36)
Vitamin D nmol/L	57,659	62 (44, 80)	58 (40, 77)	61 (44, 81)	62 (45, 80)
Vitamin B12 pmol/L	108,926	304 (247, 380)	307 (248, 386)	306 (248, 382)	309 (250, 383)
Folate nmol/L	80,919	14 (10, 20)	14 (10, 20)	14 (10, 20)	14 (10, 20)
Homocysteine µmol/L	43,186	10.4 (8.4, 12.9)	10.2 (8.2, 12.6)	10.3 (8.3, 12.7)	10.3 (8.3, 12.8)
Methylmalonic acid µmol/L	21,544	0.15 (0.12, 0.18)	0.14 (0.11, 0.18)	0.14 (0.12, 0.18)	0.15 (0.12, 0.19)
C-reactive protein high sensitivity mg/L	31,172	1.0 (1.0, 3.0)	1.0 (1.0, 3.0)	1.0 (1.0, 3.0)	1.0 (1.0, 3.0)
Sedimentation rate mm	6,461	4 (2, 8)	4 (2, 10)	5 (2, 10)	4 (1, 10)
Carbohydrate deficient transferrin %	4,902	0.80 (0.60, 1.20)	0.80 (0.70, 1.50)	0.80 (0.60, 1.30)	0.90 (0.70, 1.60)
Phosphatidylethanol µmol/L	232	0.11 (0.02, 0.40)	0.09 (0.00, 0.20)	0.24 (0.02, 0.41)	0.18 (0.02, 0.75)

Median (IQR); n (%).

Phosphatidylethanol, HbA1c and sedimentation rate was measured in whole blood, and homocysteine was measured in plasma. The first Lp(a) measurement in each individual between 2000-2019 was included. Creatinine measurements were traceable to isotope dilution mass spectrometry (IDMS). eGFR was calculated from the 2009 CKD-EPI equation. Glucose was tested while fasting. LDL-C = low-density lipoprotein cholesterol; LDL-C-corr = low-density lipoprotein cholesterol corrected; HDL-C = high-density lipoprotein cholesterol; HbA1c = haemoglobin A1c; eGFR = estimated glomerular filtration rate; TSH = thyroid stimulating hormone; IQR = interquartile range.

### 2.3 Study design and inclusion criteria

Due to changes in assays and measurement interval in reported Lp(a)-values, analyses here are presented in two periods separately. Different time periods of the Siemens assay (2009–2019) were included for the different analyses. The periods 2000-2009 and 2018-2019 were compared to analyze the proportions of individuals with Lp(a) laboratory results above clinical decision-making thresholds (50 mg/dL, 100 mg/dL and 180 mg/dL). These time periods were included due to similar measurement intervals. Sensitivity analyses were performed in individuals with measurements analyzed by both assays in 2000–2009 and 2009–2019, analyzing the proportion of individuals with Lp(a) levels above the thresholds of 50 mg/dL and 85 mg/dL. Data from 2009–2019 (Siemens assay) was used to study the distribution of Lp(a) in the study population.

In individuals with multiple Lp(a) measurements between 2000–2019, the first measurement analyzed with each assay was included in the analysis.

### 2.4 Statistical analyses

We performed all data analyses in R version 4.2.3 [16] using RStudio (Boston, MA, USA, www.rstudio.com) and the tidyverse framework, including data cleaning, data manipulation, data modeling, and data visualization [17]. The ratio between the performance of the Roche and Siemens assay was calculated as $\frac{\%\;individuals\;>Lp(a)threshold\;in\;2000-2009}{\%\;individuals\;>Lp(a)threshold\;in\;2018-2019}$.

### 2.5 Ethics and data protection

The project was approved by the Regional Committees for Medical and Health Research Ethics (REK) in Norway (ref. 2016/1693). The patient data was de-identified by Fürst Medical Laboratory in advance to transferring it to Service for sensitive data (TSD) at University of Oslo. In accordance with the EU General Data Protection Regulation, a data protection impact assessment (DPIA) was conducted and approved by the internal privacy protection deputy at the University of Oslo.

## 3 Results

Patient characteristics of this study cohort with median age of 38.8 years (inter quartile range (IQR): 31.1-45.0) are shown in Table 1.

### 3.1 Lp(a) levels in 2000 – 2019

**Fig. 1:**
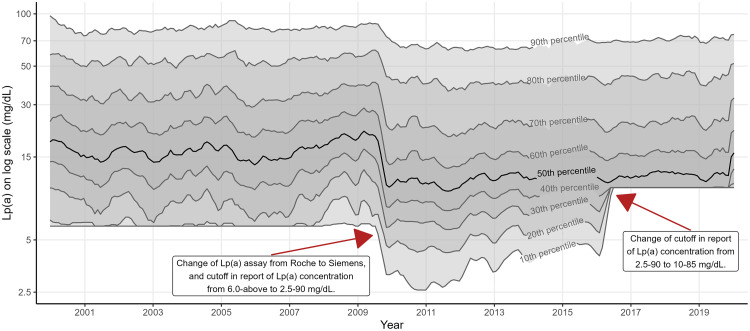
Percentiles of Lp(a) levels in serum samples between 2000 – 2019. Date of Lp(a) measurement was cut into breaks of 30 days. Rolling average of 10^th^, 20^th^, 30^th^, 40^th^, 50^th^ (median), 60^th^, 70^th^, 80^th^ and 90^th^ percentile of Lp(a) on log scale in each break was calculated. X-axis is date on continuous scale but breaks per year is shown here. Lp(a) values are on log scale, and the Y-axis has been back-transformed. Red arrows indicate the most important changes in the methods of analyzing Lp(a). Measuring interval in 2000-2009 was 6.0-354 mg/d, analyzed by the Roche Tina-quant assay. Measuring interval in 2009-2016, 2016-2018 and 2018-2019, analyzed by the Siemens Lipoprotein(a) assay, was 2.5-90 mg/dL, 10-85 mg/dL and 10-340 mg/dL, respectively. Lp(a) = lipoprotein(a).

Percentiles of Lp(a) levels are shown in Fig. 1. The change of assay in analyzing Lp(a) and measuring interval in reporting Lp(a) levels, as described in the methods section, resulted in shift of all percentiles of Lp(a) levels. The Roche Tina-quant assay (generation 1) (2000-2009) and Siemens LPA assay (2009-2019) had median Lp(a) levels of 16.8 [IQR: 7.2-44.1] and 11.3 [IQR: 8.2-30.1] mg/dL, respectively. The 50^th^, 60^th^, 70^th^, 80^th^ and 90^th^ percentile of Lp(a) in each assay, and the difference in mg/dL and % between the Roche and Siemens assay in each percentile is shown in Table S2.

### 3.2 Distribution of Lp(a) in a Norwegian cohort

**Fig. 2:**
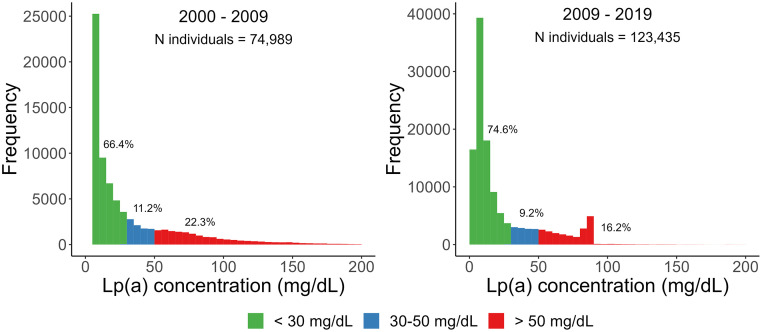
Distribution of Lp(a) in this Norwegian study cohort using common thresholds, where 50 mg/dL was determined as the 80^th^ percentile in Caucasians (1). The graph is based on serum samples from 74,989 individuals between 2000-September 2009 and 123,435 individuals between September 2009-2019 from Fürst Medical Laboratory. Lp(a) = lipoprotein(a).

74,989 individuals measured Lp(a) between 2000-2009 and 123,435 individuals measured Lp(a) between 2009-2019. In total, 22.3 % of the individuals measured between 2000-2009 and 16.2 % of the individuals measured between 2009-2019 had high levels of Lp(a) (Fig. 2), defined as >50 mg/dL [11]. Women exhibited a slightly higher percentage of individuals with Lp(a) levels exceeding 50 mg/dL compared to men, with 22.6 % (N = 8,207) versus 22.1 % (N = 8,540) in the 2000-2009 period and 16.3 % (N = 9,451) versus 16.1 % (N = 10,556) in the 2009-19 period (results not shown).

### 3.3 Impact of change in Lp(a) immunoassay on laboratory result

The proportion of individuals with Lp(a) laboratory result above specific thresholds is shown in Table 2. The Roche assay detected 20-80 % more individuals with Lp(a) levels above the suggested clinical decision values 50 mg/dL, 100 mg/dL and 180 mg/dL, respectively, compared to the Siemens assay. The difference between the performance of the Roche and Siemens assay increased with higher Lp(a) levels. When repeating the analysis with data from 2000-2009 and 2009-2019 only for individuals that had measurements analyzed by both assays, the Roche assay detected 40 % more individuals with Lp(a) >50 mg/dL than the Siemens assay (25 %, N = 3,373 vs. 18 %, N = 2,475) and 70 % more individuals with Lp(a) >85 mg/dL than the Siemens assay (11 %, N = 1,478 vs. 7 %, N = 881) (Table S3).

**Table 2:** Proportion and number of individuals with Lp(a) laboratory result ≤ 50 mg/dL, >50 mg/dL, >100 mg/dL and >180 mg/dL, and median (IQR) of Lp(a) in mg/dL.

	Roche assay		Siemens assay		
Lp(a) threshold (mg/dL)	% (N)	Lp(a) (IQR)^a^	% (N)	Lp(a) (IQR)^a^	Ratio^b^
≤ 50	78 (58,242)	11.9 (6-22.1)	82 (15,931)	10 (10-17)	1.0
>50	22 (16,747)	79.1 (63.6-106.8)	18 (3,590)	77.2 (62.2-102.2)	1.2
>100	6.6 (4,932)	128.3 (111.9-152.7)	4.9 (965)	120.8 (108.3-140.9)	1.3
>180	0.72 (543)	204.8 (188.8-231.1)	0.40 (78)	199.6 (190.3-221.4)	1.8

Values in the respective groups in 2000-2009 (Roche assay, N = 74,989 individuals) and 2018 – 2019 (Siemens assay, N = 19,521) analyzed in serum samples.

Lp(a) = lipoprotein(a). IQR = inter quartile range.

a Median (IQR) Lp(a) in mg/dL.

b Ratio calculated by % individuals below or above Lp(a) thresholds in measurements analyzed by the Roche assay, divided by % individuals below or above Lp(a) thresholds in measurements analyzed by the Siemens assay.

## 4 Discussion

### 4.1 Differences in Lp(a) laboratory results between two immunoassays

In this large real-world study, a main finding was that the change from one Lp(a) immunoassay to another, substantially influenced the number of individuals exceeding important clinical decision-making thresholds, with the highest difference at the very highest levels. The observed variations are probably due to differences in how the assays were calibrated [18]. Two studies comparing several commercially available Lp(a) immunoassays found large differences between the assays, also likely due to how the different assays are calibrated [5,6]. There is currently no gold standard material and method for immunoassays measuring Lp(a). Nevertheless, our findings shed light on how a change from one Lp(a) immunoassay to another can affect the laboratory results of an individual in a real-life setting, especially in those with Lp(a) levels close to a clinical decision-making threshold.

The challenges around developing Lp(a) assays that are reliable, precise and accurate have been extensively reviewed elsewhere [19]. Due to the remarkable size polymorphism of apo(a) and the high homology between the different KIV repeats, it is difficult to develop antibodies that recognizes only one epitope per Lp(a) particle. Since each individual carries two different isoforms with numerous possible combinations, the isoform composition in the blood sample often differs from the isoform composition in the standard curve, further complicating the accuracy of the assays [7]. The apo(a) size variability may cause an overestimation of Lp(a) concentration in individuals with large apo(a) isoforms associated with lower Lp(a) levels, and underestimation of Lp(a) concentration in individuals with small apo(a) isoforms associated with higher Lp(a) levels, and consequently a possible misclassification of cardiovascular risk [18,20].

Therefore, the 2022 EAS Consensus panel recommended that measurement of Lp(a) should be in molar units (nmol/L) if available [2]. Expressing Lp(a) concentration in mass units, ignores a potential mass difference between Lp(a) particles with varying KIV-2 repeats, post-translational modifications of apo(a) and apo B, and Lp(a) lipid content [21]. To ensure comparability between results obtained by different methods and laboratories, it is crucial to establish metrological traceability, and there are ongoing efforts to achieve this. A working group from the International Federation of Clinical Chemistry and Laboratory Medicine (IFCC) is developing a mass spectrometry-based Lp(a) reference measurement system (RMS), which is the first step towards metrological traceability. Mass spectrometry enables molecular characterization and accurate quantitation of apo(a) molar units, unaffected by apo(a) size polymorphism. Thereby, it has potential as a higher order measurement procedure, especially in reference laboratories [21]. This could provide the basis for in-vitro immunoassay manufacturers to transition from inaccurate mass units measurements to the development of commutable calibrators in molar units, independent of the provider [22].

From a clinical point of view, there are noteworthy implications of being above or below a certain threshold. These thresholds are commonly employed in risk assessment, guiding decisions on initiating preventive treatments, and may influence the future introduction and coverage of Lp(a)-lowering treatments for patients surpassing specific thresholds.

Due to the strong genetic influence and stable nature of Lp(a) [2,3], most individuals likely undergo this measurement only once. In light of our findings, we suggest that clinicians consider re-measuring Lp(a) in patients with values close to clinically relevant thresholds if the last measurement was taken many years ago and using an older assay.

A provisional solution for the lack of standardization of Lp(a) assays today and the notable variations observed across different immunoassays, could involve establishing decision thresholds in clinical laboratories based on percentiles of their specific Lp(a) assay. Consensus statements by Nordestgaard et al. from 2016 and Kronenberg et al. from 2022 recommend that assay-specific thresholds should be defined and should represent ≥80^th^ percentile of the specific Lp(a) assay [2,23]. Given the observed linear correlation between the rising concentration of Lp(a) and the estimated lifetime risk of major cardiovascular events, relying solely on one threshold, the 80th percentile estimate, appears imprecise. A value near the 80th percentile suggests an approximate 25 % heightened risk, whereas a value at the 99th percentile indicates a substantial 250 % increased estimated lifetime risk of major cardiovascular events [2]. Consequently, it is crucial to communicate risk assessments along an ordinal scale, not only dividing in low or high Lp(a) as commonly used in a historical view, applying greater precision of risk estimation with increasing elevated Lp(a) levels.

### 4.2 Study strengths and limitations

An important strength of our study is the large sample-size of our cohort of 185,493 relatively young individuals, where 75,221 and 123,824 Lp(a) measurements were analyzed by the Roche and Siemens assays, respectively.

Another strength is that all Lp(a) measurements were analyzed in fresh serum samples. The majority of the measurements (95 %) were analyzed within 24 hours, and the remaining 5 % analyzed in less than 3 days. In 1996, Kronenberg et al., showed that measurement of Lp(a) in several-year-old frozen samples is likely to result in decay and underestimated Lp(a) concentrations. The decrease became larger with a decreasing number of kringle IV repeats of apo(a), having a larger effect on individuals with high Lp(a) levels [24].

Limitations include that the data material was retrieved from patients seeking health care for unknown reasons. This could include screening for lipid disorders, patients with known health conditions or general screening, and could result in confounding by indication in our database, compared to the general population. However, the distribution of Lp(a) in this Norwegian cohort, is similar to the distribution of Lp(a) in a random and representative sample of the Danish general population [12]. Additionally, the lipid profile in our database was similar to the data in a large Norwegian population-based study from the same period of time (Table S1). Therefore, Fürst Medical Laboratory's large coverage of Lp(a) measurements in Norway and the sample-size of our cohort probably makes the present study reasonably representative for the Norwegian population.

Another limitation is that both assays used in this study expressed Lp(a) test results in mass units (mg/dL), rather than the recommended molar units (nmol/L) [2]. However, given that Lp(a) is more commonly measured in mass units than in molar units worldwide [25], our study aligns with the current clinical practices for Lp(a) measurement. It is also worth to mention that the Roche assay (Gen. 1) used in this study is no longer commercially available. Thus, we cannot determine whether the difference between the currently available assays is smaller or larger than the difference we observed between the two assays we studied.

A further limitation is the dynamic nature of the cohort considered in this study, which could introduce variability and potential bias into the data. However, due to the large number of persons examined, and that the laboratory mainly received samples from the primary health care, it is not likely that a dynamic change of the cohort influenced the Lp(a) levels by time. Additionally, when analyses were repeated using data solely from individuals that had measurements analyzed by both Roche and Siemens assays, the results were consistent (Table S3). Although such a change could not be ruled out, the distribution of Lp(a) concentrations observed, reflects what clinicians meet when assessing the person's risk profile or make the decision to retest with an improved assay.

## 5 Conclusion

In this real-world study involving 185,493 individuals, we observed that transitioning from one Lp(a) immunoassay to another affected the proportion of individuals with Lp(a) levels above clinical thresholds. The choice of assay can therefore impact an individual's laboratory results, carrying potential clinical significance, particularly for those with Lp(a) levels nearing a clinical threshold. Thus, in individuals close to relevant Lp(a) thresholds, clinicians may consider to re-measure Lp(a) particularly if the latest measurement is many years old. Our results also highlight the need of developing a reference measurement system to ensure comparability between results obtained by different methods.

## Author agreement

All authors have participated in the research reported, agreed to be an author, and read and approved submission of the manuscript. The manuscript has not been published elsewhere, nor is it under consideration elsewhere, either in part or in whole.

## Disclaimers

Torleif Trydal was previously employed and is currently receiving compensation for overseeing chemical pathology reports at Fürst Medical Laboratory.

## Declaration of generative AI in scientific writing

During the preparation of this work, the authors used GPT UiO in order to improve readability and language. After using this service, the authors reviewed and edited the content as needed and take full responsibility for the content of the publication.

## Funding sources

This work was funded by the Research Council of Norway (Oslo, Norway) (project nr. 271555/F20), and the Medical Student Research Program (MSRP) at University of Oslo (Oslo, Norway).

Novartis Pharma AG had no role in funding of this study.

## CRediT authorship contribution statement

**Janeni Jeevanathan:** Writing – review & editing, Writing – original draft, Visualization, Methodology, Funding acquisition, Formal analysis, Data curation, Conceptualization. **Sigrid M. Blom:** Writing – review & editing, Supervision, Methodology, Conceptualization. **Thomas Olsen:** Writing – review & editing, Formal analysis. **Kirsten B. Holven:** Writing – review & editing. **Erik K. Arnesen:** Writing – review & editing, Data curation. **Torleif Trydal:** Writing – review & editing, Methodology, Data curation. **Børge G. Nordestgaard:** Writing – review & editing, Methodology. **Michael Sovershaev:** Writing – review & editing. **Ying Chen:** Writing – review & editing. **Kjetil Retterstøl:** Writing – review & editing, Supervision, Methodology, Funding acquisition, Conceptualization. **Jacob J. Christensen:** Writing – review & editing, Visualization, Supervision, Methodology, Formal analysis, Data curation, Conceptualization.

## Declaration of competing interest

Jeevanathan has received consultancy fees from Novartis, and had a part time student internship at Novartis organized by the Faculty of Medicine at University of Oslo and the Student Association for Medical Innovation prior to this study.

Dr. Blom is an employee of Novartis Norway AS.

Dr. Nordestgaard has had consultancies or talks sponsored by Abbott, Akcea, Amarin, Amgen, AstraZeneca, Denka, Esperion, Kowa, Lilly, Mankind, Novartis, Novo Nordisk, Regeneron, Sanofi, Silence Therapeutics, Ultragenyx, and USV.

Dr. Retterstøl has received personal fees from Amgen, Mills AS, The Norwegian Medical Association, The Norwegian Directorate of Health, Sanofi, Novo Nordisk, none of which are related to the content of this manuscript.

The other authors have no financial relationships relevant to disclose.

## Appendix Supplementary materials

Image, application 1
