# Supplementary material for: Real-world impact of transitioning from one lipoprotein(a) assay to another in a clinical setting
Source: Am J Prev Cardiol. 2024 Aug 25;19:100726. doi: 10.1016/j.ajpc.2024.100726 (PMC11402909; doi:10.1016/j.ajpc.2024.100726)
Supplement: Supplementary file 1 [file mmc1.docx]

Supplementary materials

**Real-world impact of transitioning from one lipoprotein(a) assay to another in a clinical setting**

Janeni Jeevanathan; Sigrid M. Blom; Thomas Olsen; Kirsten B. Holven; Erik K. Arnesen; Torleif Trydal; Børge G. Nordestgaard; Michael Sovershaev; Ying Chen; Kjetil Retterstøl; Jacob J. Christensen

Contents

[**Figure S1: Users of lipid-lowering drugs (%) in individuals <50 years of age and ≥50 years of age in Norway, 2004-2019.** 2](#_Toc170660729)

[**Table S1: Mean (95% CI) total cholesterol of individuals in women and men aged 20-49 years in 2006-2008 and 2017-2019 in this study database and two large population-based cohorts in Norway** 2](#_Toc170660730)

[**Table S2: 50th, 60th, 70th, 80th and 90th percentile of Lp(a) (mg/dL) in the Roche Tina-quant assay (generation 1) (2000-2009) and Siemens LPA assay (2009-2019)** 3](#_Toc170660731)

[**Table S3: Proportion and number of individuals with Lp(a) laboratory result ≤ 50 mg/dL, >50 mg/dL, >100 mg/dL and >180 mg/dL, and median (IQR) of Lp(a) in mg/dL in individuals that had measurements analyzed by Roche and Siemens assays** 3](#_Toc170660732)

[**References** 4](#_Toc170660733)

### **Figure S1: Users of lipid-lowering drugs (%) in individuals <50 years of age and ≥50 years of age in Norway, 2004-2019.**


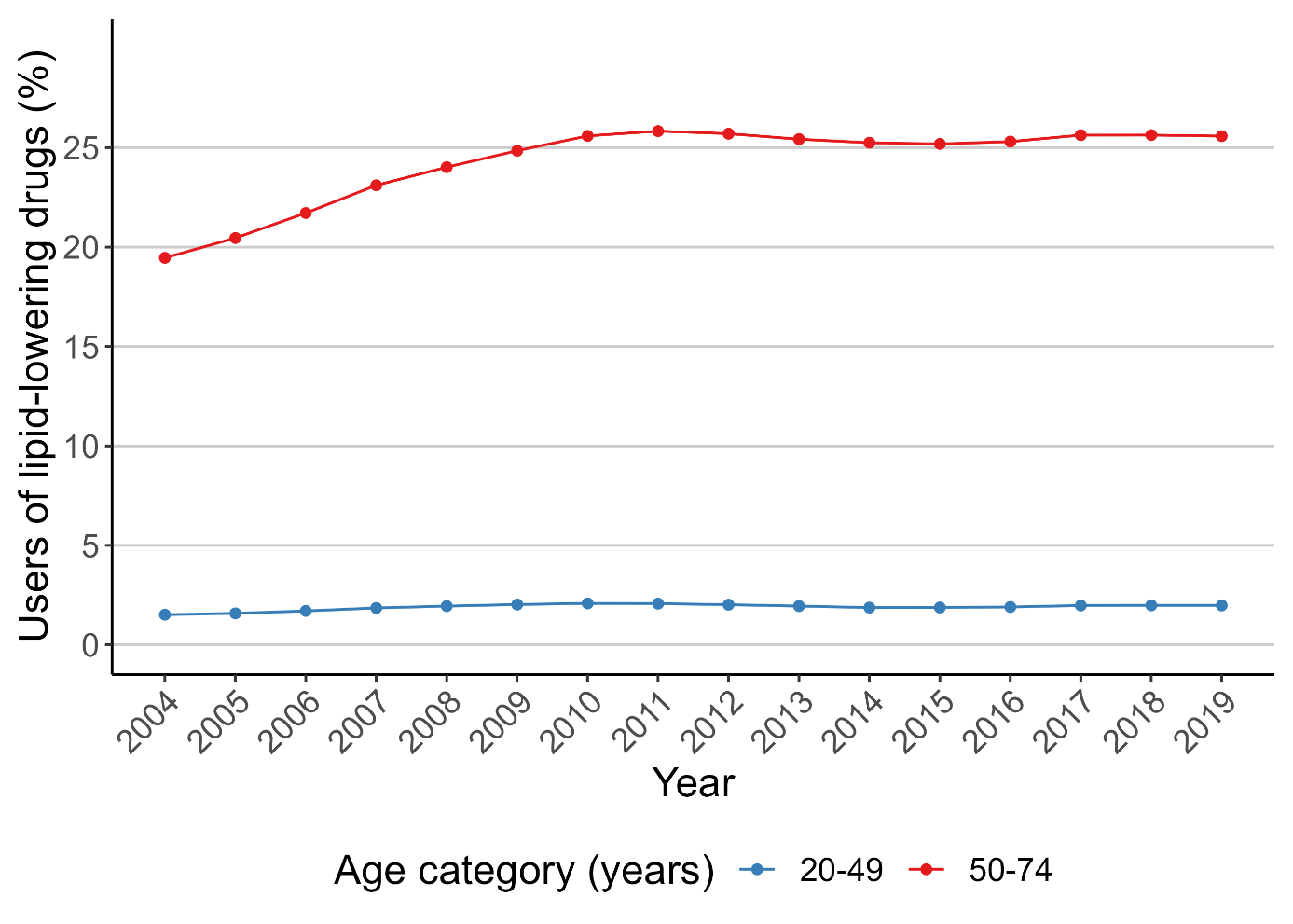


**Retrieved from the Norwegian Prescription Database, January 19th, 2024.**

### **Table S1: Mean (95% CI) total cholesterol of individuals in women and men aged 20-49 years in 2006-2008 and 2017-2019 in this study database and two large population-based cohorts in Norway**

| **Mean (95% CI) cholesterol levels in women (mmol/L)** | | | | |
| --- | --- | --- | --- | --- |
| **Age** | **HUNT**  **(2006-08)** | **Study database, Mid-Norway**  **(2006-2008)** | **Tromsø-study**  **(2007-2008)** | **Study database, Northern Norway**  **(2007-2008)** |
| 20-49 | 5.0 | 5.5 (5.3-5.6) | 5.0 | 5.3 (4.8-5.8) |
|  |  |  |  |  |
| **Mean (95% CI) cholesterol levels in men (mmol/L)** | | | | |
| **Age** | **HUNT (2006-08)** | **Study database, Mid-Norway (2006-2008)** | **Tromsø-study (2007-2008)** | **Study database, Northern Norway (2007-2008)** |
| 20-49 | 5.2 | 5.7 (5.6-5.8) | 5.4 | 5.8 (5.2-6.3) |

Mean (95% CI) cholesterol levels in the HUNT (Mid-Norway) and Tromsø-study (Northern Norway) as reported by Norwegian Institute of Public Health (1). CI = confidence interval; HUNT = Nord-Trøndelag Health study.

### **Table S2: 50th, 60th, 70th, 80th and 90th percentile of Lp(a) (mg/dL) in the Roche Tina-quant assay (generation 1) (2000-2009) and Siemens LPA assay (2009-2019)**

| **Percentile** | **Roche (mg/dL)** | **Siemens (mg/dL)** | **Difference (mg/dL)** | **Difference (%)** |
| --- | --- | --- | --- | --- |
| 50th percentile | 16.8 | 11.3 | 5.5 | 32.7 |
| 60th percentile | 23.5 | 15.1 | 8.4 | 35.7 |
| 70th percentile | 34.7 | 22.9 | 11.8 | 34.0 |
| 80th percentile | 55.7 | 41.3 | 14.4 | 25.9 |
| 90th percentile | 83.4 | 67.4 | 16.0 | 19.2 |

**Difference in mg/dL and % between the Roche assay and the Siemens assay in each percentile. Lp(a) = lipoprotein(a).**

### **Table S3: Proportion and number of individuals with Lp(a) laboratory result ≤ 50 mg/dL, >50 mg/dL, >100 mg/dL and >180 mg/dL, and median (IQR) of Lp(a) in mg/dL in individuals that had measurements analyzed by Roche and Siemens assays**

|  | **Roche assay** | | **Siemens assay** |  |  |
| --- | --- | --- | --- | --- | --- |
| **Lp(a) threshold**  **(mg/dL)** | **% (N)** | **Lp(a) concentration**  **Median (IQR)^a^** | **% (N)** | **Lp(a) concentration**  **Median (IQR)^a^** | **Ratio^b^** |
| ≤ 50 | 75 (10,121) | 12.2 (6-22.4) | 82 (11,019) | 10 (5.6-17) | 0.9 |
| >50 | 25 (3,373) | 79.5 (64.2-107.8) | 18 (2,475) | 75.6 (61-90) | 1.4 |
| >85 | 11 (1,478) | 113.4 (95.9-140.2) | 7 (881) | 90 (90-90) | 1.7 |

N individuals with Lp(a) measurements analyzed by both Roche **(2000-2009) and Siemens (2009-2019) assays 13,494.**

^a^Median (IQR) Lp(a) in mg/dL.
^b^Ratio calculated by % individuals below or above Lp(a) thresholds in measurements analyzed by the Roche assay, divided by % individuals below or above Lp(a) thresholds in measurements analyzed by the Siemens assay.
 Lp(a) = lipoprotein(a). IQR = inter quartile range.

### **References**

1. Folkehelseinstituttet [Internet]. 2021 [cited 2024 Mar 1]. Kolesterolnivå (indikator 17). Available from: https://www.fhi.no/is/ncd/kolesterol/kolesterol1/
